# Whipple Disease Initial Presentation as Supraclavicular Lymphadenopathy in a Patient With Rheumatoid Arthritis: A Diagnostic Challenge

**DOI:** 10.1177/10668969251342475

**Published:** 2025-05-21

**Authors:** Sara E. Amin, Swati Gite, Audrey Wanger, Violeta Chavez, Jacob Armstrong, Beenu Thakral, Patricia Navarro, Jennifer Makhoul, Amer Wahed, Navneet Narula, Karan Saluja

**Affiliations:** 1 Department of Pathology and Laboratory Medicine, 12340 The University of Texas Health Science Center at Houston, Houston, TX, USA; 2 Department of Hematopathology, 4002 The University of Texas MD Anderson Cancer Center, Houston, TX, USA; 3 Division of Infectious Disease, 12340 The University of Texas Health Science Center at Houston, Houston, TX, USA

**Keywords:** Whipple disease, tropheryma, lymphadenopathy, histiocytosis, electron microscopy, ultrastructure, PCR

## Abstract

Whipple disease is a rare systemic disease caused by *Tropheryma whipplei.* It can present with a wide range of nonspecific symptoms that might overlap with underlying medical conditions, posing a diagnostic challenge. We present a 61-year-old man with a past medical history of rheumatoid arthritis and chronic inflammatory demyelinating polyneuropathy, who presented with persistent leukocytosis and supraclavicular lymphadenopathy. A positron emission tomography scan revealed hypermetabolic activity in the supraclavicular, abdominal, and pelvic lymph nodes along with a myocardial hypermetabolic lesion, concerning a lymphoproliferative disorder versus sarcoidosis. A supraclavicular lymph node excisional biopsy revealed complete architectural effacement by a diffuse foamy histiocytic infiltrate without granuloma formation or necrosis. The differential diagnosis included underlying infection, histiocytic neoplasm, lymphoproliferative disorder, storage disorder, and others. Immunostains show the histiocytic cells were positive for CD68 and CD163, while negative for S100, CD1a, kappa, lambda, and BRAF V600E. Grocott-Gömöri's methenamine silver and periodic acid–Schiff (PAS) stains demonstrated diffuse intracytoplasmic granular staining that was resistant to diastase treatment. Gram, acid fast bacilli, and Fite stains were negative. Electron microscopy revealed rod-shaped organisms with a trilaminar plasma membrane, morphologically consistent with *T whipplei.* Polymerase chain reaction was positive for *T whipplei*, confirming the diagnosis. Although rare, Whipple disease should be considered in immunocompromised patients presenting with nonspecific nongastrointestinal symptoms and lymphadenopathy clinically simulating lymphoma. Periodic acid–Schiff D-positive inclusions, along with confirmatory molecular results, are crucial for diagnosis. Whipple disease is a curable disease that can be lethal if unrecognized, emphasizing the importance of heightened awareness for early diagnosis and timely treatment.

## Introduction

Histiocytic lesions encompass a wide range of underlying pathologies, including infectious, reactive, and neoplastic processes. These lesions can occur in both nodal and extranodal locations, presenting either as diffuse histiocytic infiltration or organized granulomatous inflammation.^1,2^ However, these patterns are nonspecific and do not readily lead to a definitive diagnosis. Whipple disease is one of the potential causes within this broad category. While traditionally known for its gastrointestinal manifestations, various review articles, studies, and case reports highlight Whipple disease as a “great mimicker,” with many patients overlooked due to its nonspecific clinical presentation.^3^ Moreover, although Whipple disease is a systemic disease, it can sometimes affect only a single organ for years, such as the joints or nervous system, making the diagnosis even more challenging.^4‐6^

In this report, we present a patient diagnosed with Whipple disease at our practice, featuring histiocytic supraclavicular lymphadenopathy. The patient's past medical history was significant for seronegative rheumatoid arthritis and chronic inflammatory demyelinating polyneuropathy illustrating the complex clinical presentation of Whipple disease in the context of other overlapping medical conditions and long-term iatrogenic immunosuppression.

## Patient Presentation

A 61-year-old man with a 10-year history of seronegative rheumatoid arthritis had been treated with multiple regimens, including methotrexate, adalimumab (TNF blocker), hydroxychloroquine, and low-dose prednisone. Despite treatment, he continued to experience significant morning stiffness lasting several hours, primarily affecting his shoulder and hand joints, with difficulty in gripping objects.

Two years prior to his presentation, the patient came to our hospital with worsening bilateral burning and tingling pain in his hands. A lumbar puncture revealed elevated cerebrospinal fluid protein levels and magnetic resonance imaging of the brachial plexus showed abnormal T2 signals in the right C5-C7 nerve roots. He was diagnosed with chronic inflammatory demyelinating polyneuropathy and was initially treated with intravenous immunoglobulin followed by corticosteroids. His neuropathic pain fluctuated and eventually extended to his lower extremities. Despite trials of several neuropathic medications, he experienced only partial relief.

Additionally, he reported xerostomia but denied having oral ulcers, vision changes, or dry eyes. A minor salivary gland biopsy from lower lip revealed an increased ratio of IgG4/IgG-positive plasma cells (>40%) with focal stromal fibrosis, suggesting IgG4-related disease; however, serum IgG4 levels were normal. The patient had no gastrointestinal symptoms but was found to have low-positive serum SS-B (La) antibodies with negative SS-A (Ro) antibodies, raising the suspicion for Sjögren's syndrome. As a result, Rituximab infusions and high-dose prednisone were started.

Over the past 2 years, the patient experienced persistent low-grade fever, leukocytosis, and elevated acute phase reactants, which were attributed to his underlying rheumatologic conditions. Routine follow-up imaging revealed abdominopelvic lymphadenopathy, and a positron emission tomography scan revealed hypermetabolic activity in the supraclavicular, abdominal, and pelvic lymph nodes, as well as a hypermetabolic lesion in the myocardium. These findings raised concerns about possible lymphoproliferative disorders, infection, or sarcoidosis. A comprehensive laboratory work-up including urine *Histoplasma* antigen, tuberculosis testing (QuantiFERON), serum immunofixation, blood cultures, and blood angiotensin-converting enzyme levels were negative. Subsequently, an excisional biopsy of the supraclavicular lymph node was performed for further evaluation.

## Results

**Figure 1.**
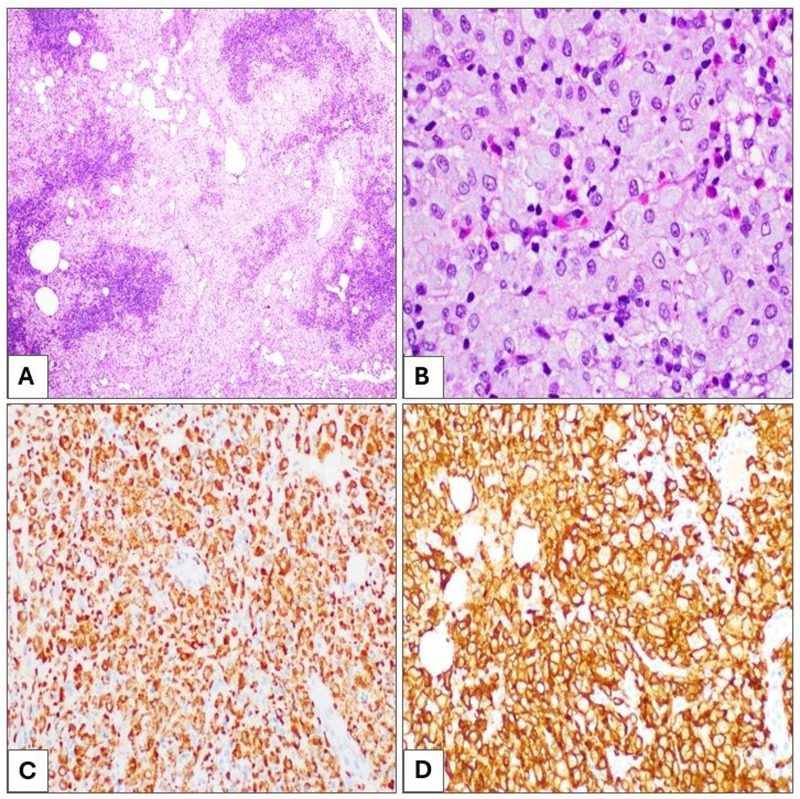
Supraclavicular lymph node tissue section showing diffuse foamy histiocytic infiltration with few scattered eosinophils, H&E (A and B); 40× and 400×, respectively. The foamy histiocytes are diffusely positive for CD68 (C) and CD163 (D) immunostains; 40×.

Histological examination of the supraclavicular lymph node revealed complete parenchymal effacement, with extensive infiltration by sheet of foamy histiocytes without definitive granuloma formation. Scattered cystic spaces were noted, surrounded by macrophages. The background contained scattered eosinophils and a few plasma cells (Figure 1A and B).

Immunohistochemical staining, performed on 4-μm thick formalin-fixed, paraffin-embedded tissue sections, showed that the histiocytes were positive for CD68 and CD163, and negative for S100, cyclin D1, CD1a, BRAF V600E, and ALK (Figure 1C and D) excluding diagnoses of Rosai-Dorfman disease (RDD), Langerhans cell histiocytosis (LCH), and ALK-positive histiocytosis. CD3 and CD20 highlighted few scattered T-cells and B-cells, respectively, excluding any lymphoproliferative neoplasm. Kappa and lambda immunostains did not show any evidence of light chain clonality, ruling out crystal-storing histiocytosis. IgG and IgG4 did not reveal increased IgG4/IgG ratio, excluding IgG4-related disease. Chromogenic in situ hybridization for Epstein-Barr virus-encoded small RNA was also negative.

**Figure 2.**
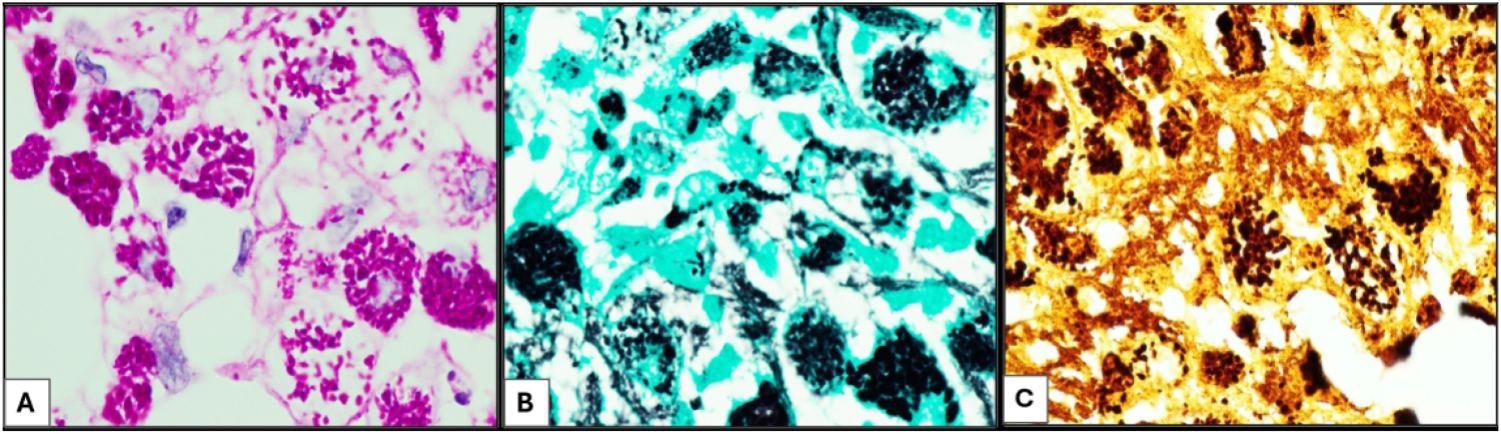
Special stains, (A) PAS stain highlighting intracytoplasmic as well as interstitial clusters of different sizes and shapes which are resistant to diastase treatment (PAS-D). These clusters are also highlighted by GMS (B) and Warthin-Starry stain (C); oil immersion at 1000× magnification.

**Figure 3.**
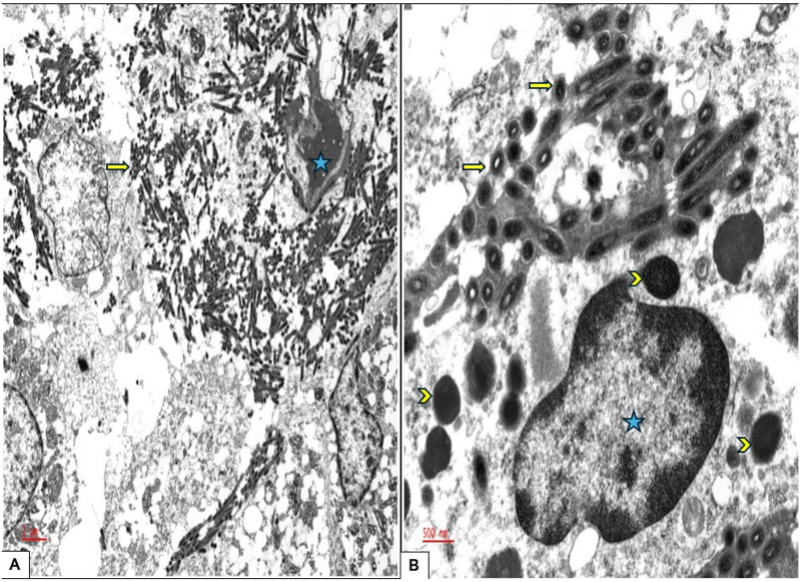
Ultrastructure electron microscopy examination, (A) Rod-shaped organisms measuring up to 2.5 microns in length and 0.2 microns in width are visible, appearing tubular in cross-section (arrow). These organisms are located within the histiocyte (the nucleus is designated with a star) as well as in the interstitial tissue; 5000×. (B) A single histiocyte is shown packed with organisms. Notably, the organisms display a characteristic trilaminar membrane (arrow), which aids in differentiating them from other intracellular organelles (arrowhead); 20 000×.

**Figure 4.**
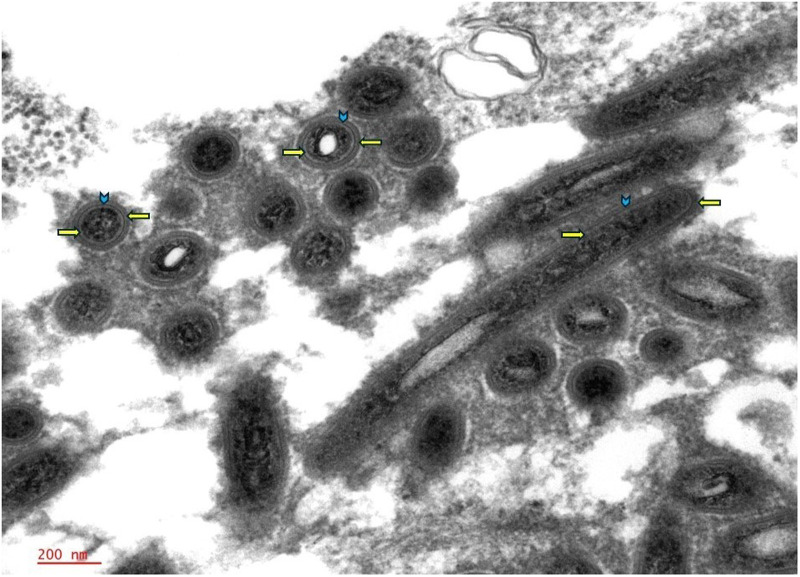
Ultrastructural examination highlighted the detailed structure of the trilaminar membrane, characterized by distinct internal and outer plasma membranes (indicated by right and left arrows, respectively) with the cell wall in between (arrowhead). This outer wall morphology is characteristic of *Tropheryma whipplei*; 50 000×.

Special stains including Grocott-Gömöri's methenamine silver (GMS), Periodic acid–Schiff (PAS), PAS with diastase (PAS-D), acid fast bacilli, Fite, Warthin-Starry, and Gram stains were performed. Within the macrophages, there were characteristic intracytoplasmic PAS-positive, diastase-resistant coarse granules, which were also highlighted by GMS and Warthin-Starry stains, suggesting an infectious etiology (Figure 2). Acid fast bacilli, Fite, and Gram stains were negative. The clusters of these coarse granules were of varying size and shape and appeared larger than any typical microorganism. Ultrastructural examination by electron microscopy revealed the presence of abundant rod-shaped organisms, measuring 1.0 to 2.5 microns in length and 0.2 to 0.3 microns in width, with the outer wall showing a trilaminar membrane (TM) morphologically consistent with *Tropheryma whipplei* (Figures 3 and 4).

The presence of *T whipplei* was confirmed by PCR testing of DNA extracts from formalin-fixed, paraffin-embedded block at a reference laboratory, utilizing seminested PCR targeting a 500 bp segment of the heat shock protein 65 (*hsp65*) gene specific for the organism.

## Discussion

Whipple disease is a rare systemic infection caused by the gram-positive bacterium *T whipplei*. This organism is widespread in the environment, commonly found in soil and sewage, and has been isolated from the stool of asymptomatic carriers. The infection often occurs in individuals with frequent exposure to animals or sewage.^7‐9^ The pathogenesis of the disease is mostly unknown, with some studies highlighting a defect in the T-helper cells as a reason for increased susceptibility, while others show that this defect is actually induced by the organism itself.^10,11^ However, as rule of thumb, macrophages are unable to digest the organism. The rarity of the disease, despite the presence of asymptomatic carriers, suggests that immunosuppression may play a role in its development. Additionally, Whipple disease tends to run in families, and the majority of affected individuals are of European ancestry, particularly males. Although intrafamilial circulation may contribute to the higher prevalence in these families, the role of genetic predisposition remains unclear.^7,12,13^

Whipple disease can affect any organ system and presents with a wide range of clinical manifestations, from asymptomatic carriers to potentially life-threatening conditions.^4^ Initially recognized for its gastrointestinal symptoms, such as diarrhea, abdominal pain, and malabsorption, Whipple disease is now considered a “great mimicker.”^3,4^ Many patients complain primarily of a nonspecific joint pain, leading to misdiagnoses like seronegative rheumatoid arthritis, often without any gastrointestinal symptoms.^6^ Whipple disease can also involve a single organ system, such as the heart, where it may present as culture-negative endocarditis. In a minority of patients, neurologic symptoms occur, including burning and tingling sensations, along with musculoskeletal dysfunction, such as muscle spasms.^5,14,15^

Lymphadenopathy as a presenting symptom has been reported in some patients. While mesenteric lymph nodes are most commonly involved, generalized peripheral lymphadenopathy and supraclavicular lymphadenopathy are less frequently observed.^16‐19^ Clinicians and pathologists should be aware of these atypical, nongastrointestinal presentations to ensure timely diagnosis and appropriate treatment.

The diagnosis of Whipple disease is challenging, particularly in the absence of clinical suspicion. PCR is the most specific diagnostic tool for identifying infectious disease and detection of *T whipplei* 16S rRNA and *hsp65* gene by PCR helps to confirm the diagnosis. Accordingly, PCR is more often positive in tissues from clinically affected organs such as synovial tissue (with arthritis), the small bowel (with gastrointestinal symptoms), the endocardium (with endocarditis), or cerebrospinal fluid (with meningitis). In localized forms of Whipple disease, PCR testing of tissues from clinically unaffected areas typically yields negative results.^20^ Immunohistochemical stain of tissue specimen utilizing polyclonal rabbit antibody against *T whipplei* has shown high specificity and sensitivity for detecting, however, due to the overall rarity of the disease, it might have limited availability.^21,22^

A hallmark of histopathologic examination in Whipple disease is foamy histiocytic infiltration, which requires careful exclusion of a broad range of differential diagnoses. These include infectious agents, hematopoietic disorders or neoplasms, reactive processes, and other conditions. Table 1 provides a summary of the potential causes, organ involvement, and key immunohistochemical stain findings for various conditions associated with histiocytic infiltration.

**Table 1.** Clinical Presentation, Organ Involvement, and Work-up for Differentiating Selective Histiocytic Lesions.

	Histiocytic lesions	Clinical presentation/organ involvement	Pertinent microscopic findings	Pertinent stains	Additional studies
Infectious	Mycobacteria	Chronic cough, night sweat, WL, LAD, systemic	Necrotizing granulomatous inflammation	AFB(+)	QuantiFERON test, culture PCR
	Whipple disease	Diarrhea, WL, joint pain, burning and tingling sensation	Infiltrate of foamy histiocytes	PAS/PAS-D(+), GMS(+), Warthin-Starry(+)	PCR EM (Trilaminar membrane)
	Histoplasmosis	Asymptomatic, fever, cough, HSM, LAD, systemic (immunosuppression)	Infiltrate of foamy histiocytes	GMS(+), PAS(+)	Urine *Histoplasma* antigen test PCR
Reactive/Neoplastic	Rosai-Dorfman disease	Painless LAD, WL, fever, site-specific symptoms in extranodal disease	Large histiocytes with abundant pale cytoplasm, emperipolesis	S100(+), CD68(+), CD163(+), OCT2(+), cyclinD1(+) CD1a(-), Langerin(-), ALK(-)	
	Crystal-storing histiocytosis	Bone marrow involvement, LAD, associated with plasma cell neoplasm	Histiocytic infiltrate filled with polarizable crystals	CD68(+), CD163(+), kappa/lambda restriction	Serum and urine protein electrophoresis Immunofixation
	Langerhans cell histiocytosis	Bone lesions, skin rash, pituitary gland involvement, LAD	Reniform nuclei, eosinophilic cytoplasm, background eosinophils	CD1a(+), S100(+), CD68 (+, dot-like), Langerin(+), BRAF(+) ALK(-)	EM (Birbeck granules)
	ALK-positive histiocytosis	LAD, HSM, bone and skin lesions	Large histiocytes with abundant eosinophilic cytoplasm, vesicular nuclei, emperipolesis	CD68(+), CD163(+), ALK(+) CD1a(-), Langerin(-)	
	Erdheim-Chester disease	Bone pain, pituitary gland involvement (diabetes insipidus)	Foamy histiocytes, Touton giant cells, fibrosis, mixed inflammatory cells	CD68(+), CD163(+), BRAF(+/-), Factor XIIIa(+) CD1a(-), Langerin(-)	Imaging (“hairy kidneys,” “coated aorta,” symmetric LE osteosclerosis)
Storage disorder	Niemann-Pick disease	HSM, seizures, neurologic degeneration, interstitial lung disease	Lipid laden macrophages in various tissues	PAS(+), CD68(+)	EM (Zebra bodies) Sphingomyelinase enzyme assay
	Gaucher's disease	HSM, neurologic impairment, eye damage, bone pain and necrosis	Histiocytes with “crinkled paper” appearance of cytoplasm	PAS(+), CD68(+)	EM, Glucocerebrosidase enzyme assay
	Fabry disease	ESRD, ventricular hypertrophy, arrhythmia, GI disturbance, neuropathy, stroke	Glycosphingolipid-laden macrophages in various tissues	PAS/PAS-D(+), CD68(+)	EM (Zebra bodies) α-galactosidase A enzyme assay
Others	Sarcoidosis	Bilateral hilar LAD, systemic multiorgan involvement	Non-necrotizing granulomas with epithelioid histiocytes, multinucleated giant cells	Diagnosis of exclusion; negative for all stains	Blood ACE activity
	Metastasis carcinoma	History of known malignancy	Metastatic tumor with reactive histiocytic infiltrate	AE1/AE3(+)	Organ-specific IHC

Abbreviations: ACE, angiotensin-converting enzyme; AFB, acid fast bacilli; EM, electron microscopy; ESRD, end-stage renal disease; GMS, Grocott-Gömöri's methenamine silver; HSM, hepatosplenomegaly; IHC, immunohistochemistry; LAD, lymphadenopathy; LE, lower extremity; PAS, periodic acid–Schiff; PAS-D, periodic acid–Schiff with diastase; WL, weight loss.

A diverse array of infectious agents can result in histiocytic infiltration, including *Mycobacterium* species (*M. tuberculosis, M. genavense, M. avium* complex), *Histoplasma capsulatum, Leishmania donovani, Bartonella*, and *Toxoplasma*, among others. While necrotizing granulomatous inflammation is the dominant pattern in many infectious conditions, the absence of necrosis has been observed in some lesions, such as toxoplasmosis. Initial laboratory work-up can help narrow down the diagnosis of infectious causes, including tests such as urine *Histoplasma* antigen, QuantiFERON test for *Mycobacterium tuberculosis*, and culture studies. GMS staining can detect polysaccharide material, either in the cell wall of microorganisms or in inclusion diseases. However, it lacks specificity, and morphologic identification can be challenging. Periodic acid–Schiff staining is useful for further characterizing inclusion material, distinguishing between glycogen (which is digested by diastase) and glycopolysaccharides or glycolipids (which retain the stain after diastase digestion). While PAS-D can provide additional diagnostic clarity for infectious diseases, many storage diseases also fall into this category, emphasizing the importance of clinical and pathological correlation to guide subsequent steps.

Among the reactive and neoplastic causes of histiocytic lymphadenopathy are RDD, LCH, and Erdheim-Chester disease, all of which are rare disorders with systemic symptoms and multi-organ involvement (Table 1). Rosai-Dorfman disease is a histiocytic disorder characterized by large histiocytes with abundant pale cytoplasm that often engulfs intact lymphocytes, neutrophils, or plasma cells in a process known as emperipolesis. Rosai-Dorfman disease histiocytes are typically positive for CD68, CD163, and S100, while negative for CD1a and langerin. In contrast, LCH and Erdheim-Chester disease are neoplastic histiocytic disorders driven largely by the *BRAF* V600E mutation. Langerhans cells in LCH have a distinctive reniform (kidney-shaped) nucleus with a longitudinal nuclear groove and abundant eosinophilic cytoplasm, and the background tissue often shows increased eosinophil and plasma cell infiltrates. Erdheim-Chester disease, on the other hand, presents as a xanthogranulomatous process with abundant foamy histiocytes, scattered lymphoplasmacytic infiltrates, and Touton-like giant cells, with variable degrees of fibrosis depending on the age of the lesion. Langerhans cell histiocytosis is characteristically positive for langerin, CD1a, and S100 and may show variable or dot-like positivity for CD68. Erdheim-Chester disease, on the other hand, is positive for CD68 and CD163, may express factor XIIIa, and is negative for langerin and CD1a; S100 may also be expressed.^1,2^

Lymphadenopathy in middle-aged patients often requires ruling out lymphoproliferative disorders. Flow cytometry and immunohistochemical stains for B-cell and T-cell markers and light chain restriction (kappa and lambda) are essential diagnostic tools to assess for hematologic malignancies. Crystal-storing histiocytosis is a reactive histiocytic disorder characterized by the intralysosomal accumulation of immunoglobulin, typically seen in patients with plasma cell neoplasm. These crystals will demonstrate either kappa or lambda light chain restriction, serving as a diagnostic clue to an underlying plasma cell neoplasm.^23^

Ultrastructural examination by electron microscopy, when available, is helpful in identifying the distinctive morphology of *T whipplei*, which can be seen within the epithelial cells, histiocytes, or interstitially.^24^ The bacilli appear rod-shaped, with an average width of 0.2 µm and length of 1.5 to 2.5 µm with a TM. The TM refers to the distinct structure observed in electron microscopy, comprised of an inner phospholipid bilayer that forms the inner plasma membrane, a middle peptidoglycan layer that constitutes the cell wall, and an outermost phospholipid bilayer, known as the outer plasma membrane. While this outer membrane is characteristic of gram-negative bacteria, *T whipplei,* although classified as gram-positive, exhibits structural similarities to gram-negative bacteria, including a complex cell envelope.^24^

Under electron microscopy, this TM structure presents as 2 dark lines; representing the inner and outer plasma membranes, separated by a lighter line of the peptidoglycan layer (Figures 3 and 4). This distinctive appearance is crucial for identifying the organism, aiding in differentiating it from other intracellular components and serving as a key diagnostic feature of Whipple disease by electron microscopy.

Treatment requires long-term antibiotic therapy, with treatment options including penicillin, tetracycline, and co-trimoxazole. It is worth noting that discontinuing treatment, like any other bacterial infections, can result in relapse. Interestingly hydroxychloroquine, which is frequently used in rheumatologic conditions, is currently recognized as an effective treatment of Whipple disease. It enhances antibiotic and bactericidal activity against the bacteria by altering the macrophage intraphagosomal pH. Accordingly, including hydroxychloroquine in treatment regimen is a current recommendation.^25^ Whipple disease is curable, with most extraintestinal symptoms typically resolving within days of initiating treatment.^7^ Arthritis usually resolves completely within an average of 12 months.^6^ Gastrointestinal symptoms and malnutrition, however, often take several months to improve. Neurologic manifestations, if present, tend to only partially resolve.^7^

Our patient presented a significant diagnostic challenge, enduring a 10-year history of seronegative rheumatoid arthritis, partially controlled by multiple trials of immunomodulatory and immunosuppressive medications. Throughout this period, he experienced nonspecific symptoms often attributed to “flares” of his underlying condition, which were managed by increasing corticosteroids and further immunosuppression. The onset of chronic inflammatory demyelinating polyneuropathy, accompanied by persistent leukocytosis over 2 years, was also considered part of his rheumatologic disease. Notably, he never exhibited gastrointestinal symptoms and maintained a stable body weight. Lymphadenopathy became the first concerning manifestation that prompted additional investigation.

Ultimately, the identification of PAS-D-resistant inclusions within histiocytes, the presence of a distinctive trilaminar plasma membrane upon ultrastructure electron microscopy, and confirmatory PCR testing led to the diagnosis of Whipple disease. The patient was promptly initiated on an induction regimen of ceftriaxone for 2 weeks, followed by maintenance therapy with sulfamethoxazole–trimethoprim for 2 years.

As we reflect on this patient, the question remains: Was Whipple disease the underlying cause of what appeared to be “rheumatologic conditions,” or was it a consequence of long-term immunosuppression? At the 4-month follow-up after initiating medication, the patient reported an improvement in his overall condition, although mild joint pain persisted. Notably, he did not develop any gastrointestinal symptoms throughout the course of treatment. Long-term follow-up will be crucial in answering this question. If the patient achieves a complete response to antibiotic therapy without relapse following the cessation of treatment, it will strongly support the diagnosis of Whipple disease arthritis.

## Conclusion

The perceived rarity of Whipple disease may be due to underdiagnosis or misdiagnosis, particularly as rheumatologic conditions. Additionally, the use of immunosuppressive therapies for treating rheumatologic symptoms may exacerbate the infection, leading to the clinical presentation of Whipple disease. Beyond the well-known gastrointestinal symptoms, clinicians and pathologists should be mindful of atypical presentations of Whipple disease and maintain a low threshold for suspicion. Histologic identification of GMS and PAS/PAS-D-positive rods provides valuable diagnostic clues, while confirmatory PCR testing remains the gold standard for a definitive diagnosis. Whipple disease is a curable condition that may lead to significant morbidity and even mortality if left undiagnosed, underscoring the importance of early recognition and treatment.
